# Association between embolic agent choice and complications after transcatheter arterial embolization for colonic diverticular bleeding

**DOI:** 10.1007/s00330-025-12210-y

**Published:** 2025-12-19

**Authors:** Takatoshi Kubo, Hayato Yamana, Shotaro Aso, Hiroki Matsui, Kiyohide Fushimi, Hideo Yasunaga, Osamu Abe

**Affiliations:** 1 Department of Radiology, The University of Tokyo Hospital, Bunkyo-ku, Japan; 2 Data Science Center, Jichi Medical University, Shimotsuke, Japan; 3 Department of Clinical Epidemiology and Health Economics, School of Public Health, The University of Tokyo, Bunkyo-ku, Japan; 4 Department of Real-world Evidence, Graduate School of Medicine, The University of Tokyo, Bunkyo-ku, Japan; 5 Department of Health Policy and Informatics, Graduate School of Medical and Dental Sciences, Institute of Science Tokyo, Bunkyo-ku, Japan

**Keywords:** Colonic diseases, Gastrointestinal hemorrhage, Embolization, Therapeutic

## Abstract

**Objectives:**

Although various embolic agents are used for transcatheter arterial embolization (TAE) of colonic diverticular bleeding (CDB), comparative outcome data for different embolic agents are limited. We aimed to assess the association between embolic agent choice and early rebleeding and intestinal ischemia after TAE for CDB.

**Materials and methods:**

We conducted a nationwide retrospective cohort study using the Japanese Diagnosis Procedure Combination database between July 2010 and March 2022. Adults who underwent a first TAE for CDB with coils, gelatin sponge (GS) particles, or n‑butyl‑2‑cyanoacrylate (NBCA) were included. Multivariate logistic regression analyses were performed to evaluate the association of embolic agent choice with early rebleeding requiring intervention and intestinal ischemic complications, while adjusting for covariates and within-hospital clustering.

**Results:**

The cohort comprised 5625 patients (mean age 72 years ± 12 [standard deviation], 4020 men). Coils, GS particles, and NBCA were used in 59%, 30%, and 11%, respectively. The overall early incidence of rebleeding and intestinal ischemia was 12% and 1.0%, respectively. With coils as the reference, the adjusted odds ratio for GS particles was 1.38 (95% CI: 1.15–1.66; *p* = 0.001) for early rebleeding and 2.64 (95% CI: 1.43–4.90; *p* = 0.002) for intestinal ischemia, and those for NBCA were 0.69 (95% CI: 0.50–0.95; *p* = 0.03) for early rebleeding and 3.53 (95% CI: 1.72–7.22; *p* = 0.001) for intestinal ischemia.

**Conclusions:**

Compared with coils, GS particles were associated with an increase in both early rebleeding and intestinal ischemia, whereas NBCA was associated with decreased rebleeding and increased ischemia.

**Key Points:**

***Question*** *Transcatheter arterial embolization (TAE) is one of the mainstay treatments for colonic diverticular bleeding, but outcome differences among embolic agents remain unclear.*

***Findings*** *Gelatin sponge particles increased both early rebleeding and intestinal ischemic risks, while coils reduced intestinal ischemic risk and n‑butyl‑2‑cyanoacrylate reduced early rebleeding risk.*

***Clinical relevance*** *Embolic material selection should be individualized for TAE in colonic diverticular bleeding. Coils may be safer in ischemia-prone patients, while n‑butyl‑2‑cyanoacrylate may be suitable for those at high risk of rebleeding.*

**Graphical Abstract:**

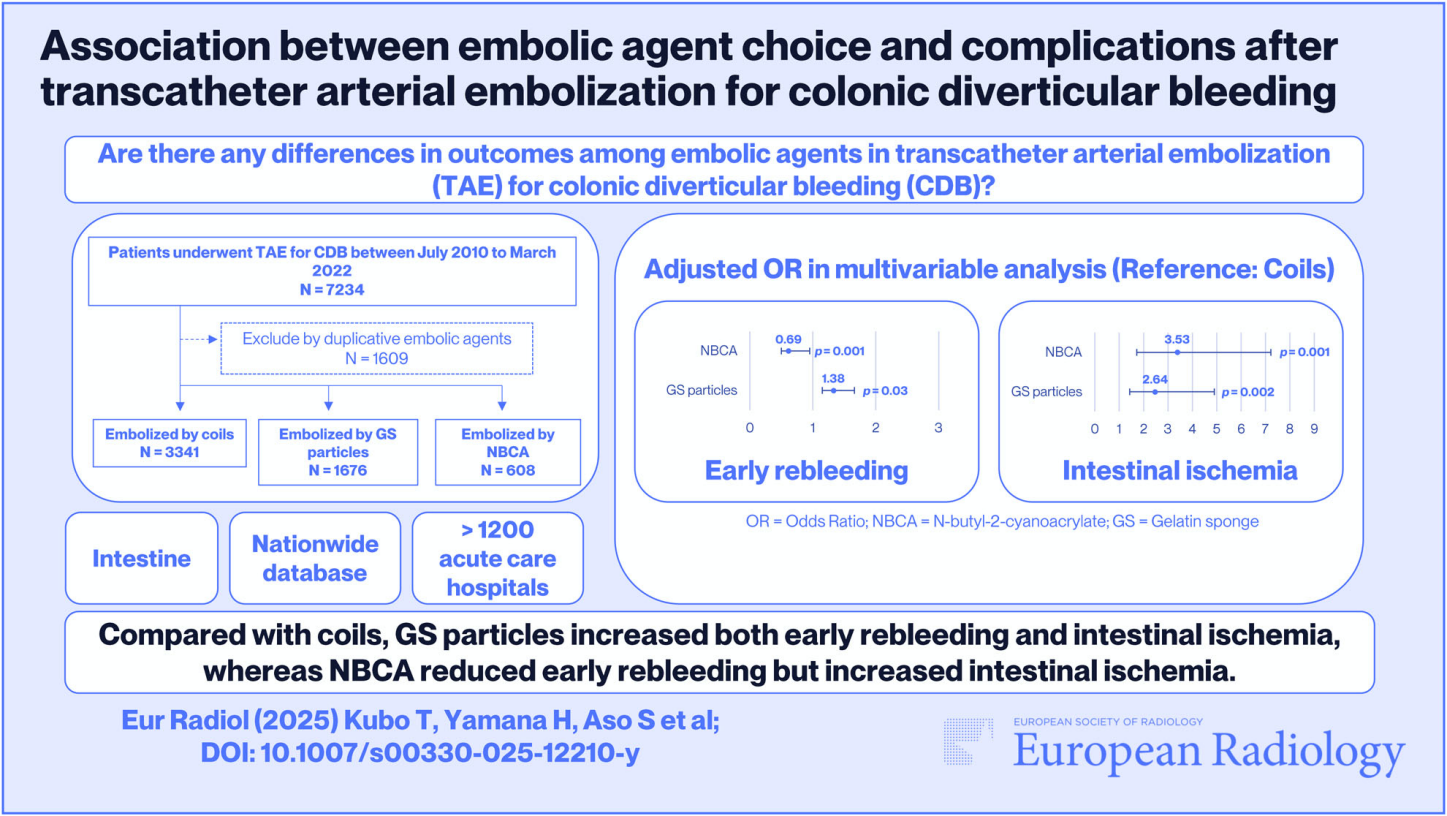

## Introduction

The incidence of colonic diverticula is increasing over time worldwide [1]. Based on 28192 colonoscopy examinees in Japan, colonic diverticula were detected in 20.3% [1]. According to a cohort study in the United States, the cumulative bleeding rate among colonic diverticulum carriers is 0.6% per year [2]. Although spontaneous hemostasis of colonic diverticular bleeding (CDB) occurs in about 70% of patients [3], hemostatic treatment is required when bleeding persists.

Hemostatic procedures for CDB include endoscopic hemostasis, transcatheter arterial embolization (TAE), and surgery. The Japan Gastroenterological Association guidelines for CDB recommend TAE for massive bleeding that cannot be controlled by colonoscopy, as well as for continuous or recurrent bleeding in which the site of bleeding cannot be identified by colonoscopy [4]. The British Society of Gastroenterology guidelines for acute lower gastrointestinal bleeding (LGIB) state that TAE can be performed if extravasation is evident on angiography [5].

Despite these recommendations, limited case series have been reported regarding TAE for CDB [6–8]. Moreover, although rebleeding and complications such as intestinal ischemia, vascular injury, and infection may occur after TAE, their precise incidence rates have not been assessed. In addition, various embolic materials such as coils, gelatin sponge (GS) particles, and n-butyl cyanoacrylate (NBCA) are used in TAE [9]. However, to the best of our knowledge, there have been no studies examining the differences in the proportions of early rebleeding and intestinal ischemia according to the embolic materials used in TAE for CDB.

Therefore, we conducted the present study using a nationwide inpatient database in Japan to investigate differences in the incidence of early rebleeding and intestinal ischemic complications after TAE for CDB using three different embolic agents: coils, GS particles, and NBCA.

## Materials and methods

The retrospective cohort study was approved by the institutional review board, and informed consent was waived due to the use of anonymized data. This study adhered to the Strengthening the Reporting of Observational Studies in Epidemiology (STROBE) guidelines.

### Data source

We used the Japanese Diagnostic Procedure Combination database, an inpatient database of discharge abstracts and administrative claims data obtained from more than 1200 acute care hospitals across Japan. This database covers 90% of all tertiary care emergency hospitals in Japan. The stored data included age, sex, body weight and height, diagnosis (including the main diagnosis, comorbidities present at admission, and complications arising after admission), procedures, prescriptions, and discharge status. Diagnoses were recorded using the International Statistical Classification of Diseases 10th Revision (ICD-10) codes and text in Japanese. Diagnostic records are linked to a payment system, and attending physicians must report accurate diagnoses for cost reimbursement [10]. Moreover, a previous study reported the validity of the database [11], with the specificity of diagnoses exceeding 96% and a sensitivity of 50–80%. Both the specificity and sensitivity of procedures exceeded 90% [11].

### Study population

Using the Japanese Diagnostic Procedure Combination database, we identified all patients aged ≥ 18 years who underwent TAE during hospitalization between July 2010 and March 2022 with the main or most resource-consuming diagnoses of colonic diverticula (ICD-10 code, K57). The first TAE during hospitalization was analyzed, and the embolic materials used on the day of the first TAE were identified. Patients who received more than one agent on the same day were excluded.

To compare the incidence of early rebleeding and intestinal ischemia after TAE according to the embolic agent, we classified the patients into three groups: those who underwent TAE with coils alone (coil group), those who underwent TAE with NBCA alone (NBCA group), and those who underwent TAE with GS particles alone or had no billing data for any embolic agent (GS particle group). In Japan, the GS particles are supplied in four forms. However, data on Gelform (Pfizer Japan) and Spongel (Astellas Pharma), which are not licensed by the national health insurance, could not be obtained. Moreover, Gelpart and Serescure (both Nippon Kayaku) were not licensed as vascular embolic agents until 2005 and 2013, respectively. Therefore, based on a previous study that used the same database [12], we categorized patients who underwent TAE without billing data for embolic agents into the GS particle group.

### Definition of early rebleeding

We defined patients with early rebleeding requiring intervention as those requiring additional TAE, endoscopic hemostasis, or surgical hemostasis within 30 days of the initial TAE.

### Definition of intestinal ischemic complications

We defined intestinal ischemic complications using the ICD-10 code K55.0 (acute vascular disorders of intestine) or K63.1 (perforation of intestine (nontraumatic)) recorded as complications arising after admission.

### Variables

Collected data included age, sex, body mass index, smoking history, admission due to CDB, type of admission, ambulance use, teaching hospital, Charlson Comorbidity Index [13] (scored based on each patient’s diagnoses), examinations before TAE, treatments before TAE, medications before TAE, use of microcatheters, intensive care unit admission, in-hospital mortality, length of hospital stay, and total hospitalization cost. We also summarized the number of TAEs performed at the hospital during the same fiscal year (hospital volume).

### Statistical analysis

All analyses were performed using the STATA/SE software (version 18.0; Stata Corp).

Patient characteristics and outcomes were summarized according to the embolic agents used. We performed bivariate and multivariable logistic regression analyses fitted with generalized estimating equations to adjust for multiple covariates and within-hospital clustering. Different models corresponding to two outcomes (early rebleeding and intestinal ischemic complications) were used. We set a hierarchical structure at the level of each institution using exchangeable correlation matrices. Binomial distribution and logit link function were specified to describe the effect estimates using odds ratios (ORs) with 95% CI. In addition to the embolic agents, the following explanatory variables were incorporated into the models based on previous studies [1, 14–16]: age, sex, body mass index (< 18.5, 18.5–24.9, ≥ 25.0 kg/m2 or missing data), smoking history (nonsmoker, current and/or past smoker or missing data), Charlson Comorbidity Index (0, 1 or ≥ 2), admission due to CDB, type of admission (scheduled or emergency), ambulance use, teaching hospital, use of microcatheters and hospital volume (1, 2, 3–4, or ≥ 5). The following procedures were also performed before TAE during hospitalization: contrast-enhanced CT, angiography, endoscopy, endoscopic hemostasis, transfusion, non-steroidal anti-inflammatory drug use, and antiplatelet or anticoagulant use.

All *p*-values were two-tailed, and *p* < 0.05 was considered statistically significant.

## Results

### Patient characteristic

**Fig. 1:**
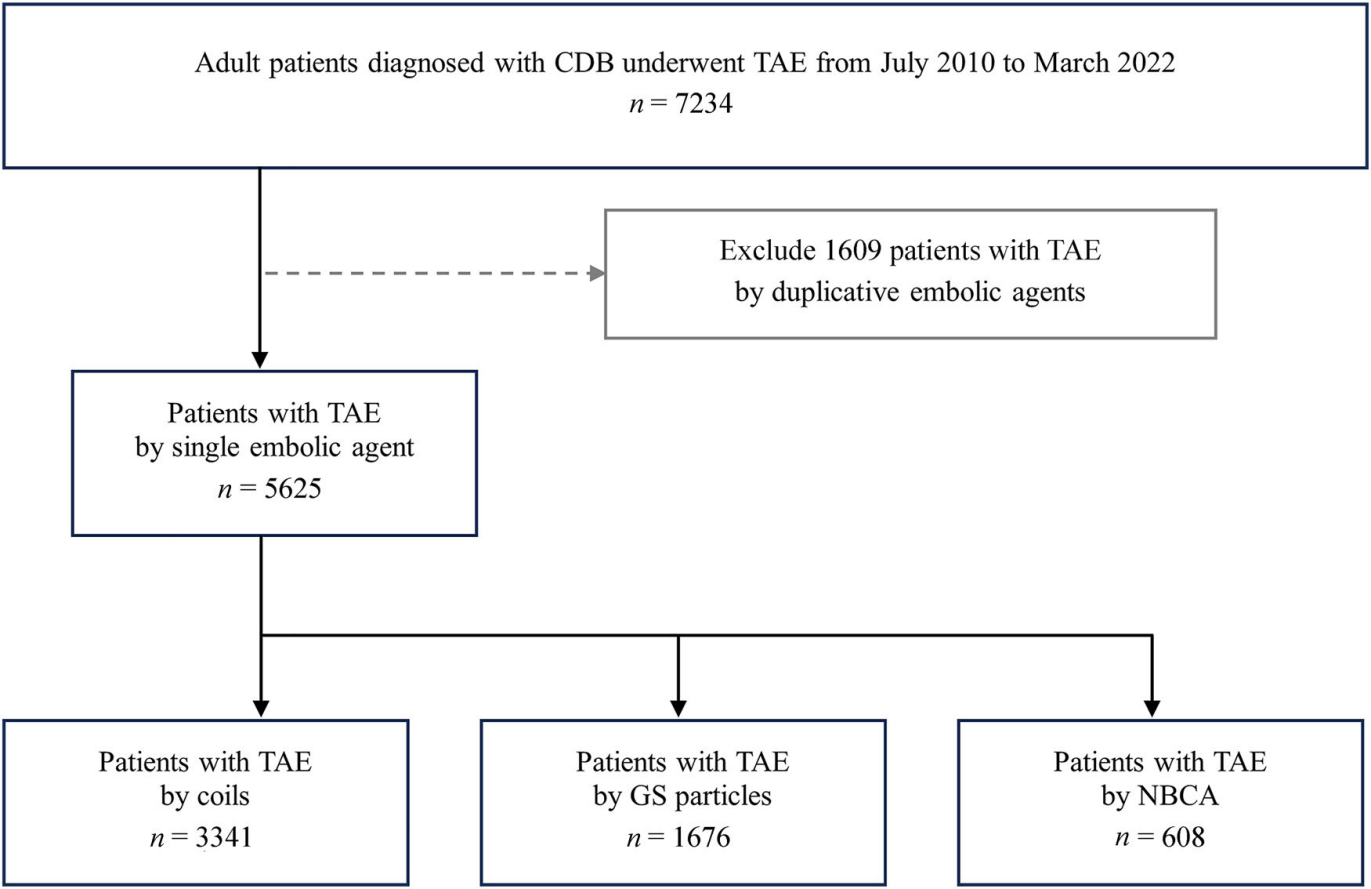
Patient selection flowchart. CDB, colonic diverticular bleeding; TAE, transcatheter arterial embolization; GS, gelatin sponge; NBCA, n-butyl-2-cyanoacrylate

We identified 7234 patients who underwent TAE for CDB. Among them, 1609 patients had been administered more than one embolic agent; therefore, 5625 patients were included in the study. Their mean age was 72 years (standard deviation, 12 years), and 4020 (71%) of them were men. Among the eligible patients, the coil, GS particle, and NBCA groups comprised 3341 (59%), 1676 (30%) and 608 (11%) patients, respectively (Fig. 1).

Table 1 shows the patient characteristics. The overall proportions of patients with admission due to CDB, emergency admission and ambulance transport were 65%, 90%, and 42%, respectively. In total, 4439 (79%), 154 (3%), and 1884 (33%) patients underwent contrast-enhanced CT, angiography, and colonoscopy before initial TAE, respectively. Colonoscopic hemostasis and transfusion were performed in 1797 (32%) and 3877 (69%) patients, respectively. Non-steroidal anti-inflammatory drugs were used in 10%, and antiplatelets or anticoagulants were used in 11%. Microcatheters were used in 96%. The in-hospital mortality rate was 3%.

**Table 1:** Patient characteristics

Parameter	All patients (*n* = 5625)	Coil group (*n* = 3341)	GS particle group (*n* = 1676)	NBCA group (*n* = 608)
Age (years)*	72 ± 12	72 ± 12	72 ± 12	73 ± 11
Male	4020 (71)	2425 (73)	1175 (70)	420 (69)
Body mass index (kg/m^2^)				
< 18.5	447 (8)	247 (7)	142 (9)	58 (10)
18.5–24.9	3286 (58)	1938 (58)	987 (59)	361 (59)
≥ 25.0	1463 (26)	913 (27)	406 (24)	144 (24)
Missing data	429 (8)	243 (7)	141 (8)	45 (7)
Smoking history				
Nonsmoker	2991 (53)	1754 (53)	925 (55)	312 (51)
Current and/or past smoker	1829 (33)	1119 (33)	501 (30)	209 (34)
Missing data	805 (14)	468 (14)	250 (15)	87 (14)
Admission due to CDB	3644 (65)	2267 (68)	963 (57)	414 (68)
Emergency admission	5042 (90)	3112 (93)	1394 (83)	536 (88)
Ambulance transportation	2372 (42)	1459 (44)	620 (37)	293 (48)
Teaching hospital	4974 (88)	2970 (89)	1465 (87)	539 (89)
Charlson comorbidity index^†^				
0	3685 (66)	2305 (69)	993 (59)	387 (64)
1	519 (9)	326 (10)	124 (7)	69 (11)
≥ 2	1421 (25)	710 (21)	559 (33)	152 (25)
Examination before TAE				
CT	4921 (87)	2991 (90)	1403 (84)	527 (87)
Contrast-enhanced CT	4439 (79)	2729 (82)	1218 (73)	492 (81)
Angiography	154 (3)	88 (3)	45 (3)	21 (3)
Colonoscopy	1884 (33)	1103 (33)	593 (35)	188 (31)
Treatment before TAE				
Colonoscopic hemostasis	1797 (32)	1151 (34)	475 (28)	171 (28)
Transfusion	3877 (69)	2384 (71)	1066 (64)	427 (70)
Medication before TAE				
Non-steroidal anti-inflammatory drugs	563 (10)	302 (9)	202 (12)	59 (10)
Antiplatelet and/or anticoagulant	592 (11)	361 (11)	154 (9)	77 (13)
Use of microcatheters	5390 (96)	3242 (97)	1564 (93)	584 (96)
Intensive care unit admission	1171 (21)	698 (21)	311 (19)	162 (27)
In-hospital mortality	149 (3)	84 (3)	37 (2)	28 (5)
Length of hospital stay (days)*	21 ± 24	20 ± 23	23 ± 25	23 ± 29
Total hospitalization cost (USD)*	11,733 ± 11,841	11,735 ± 11,003	11,609 ± 13,092	12,065 ± 12,637
Hospital volume^‡^				
1	1438 (26)	785 (24)	493 (29)	160 (26)
2	1182 (21)	686 (21)	375 (22)	121 (20)
3–4	1439 (26)	844 (25)	460 (27)	135 (22)
≥ 5	1566 (28)	1026 (31)	348 (21)	192 (32)

Unless otherwise indicated, data are presented as numbers of patients, with percentages in parentheses

*GS* gelatin sponge, *NBCA* n-butyl-2-cyanoacrylate, *CDB* colonic diverticular bleeding, *TAE* transcatheter arterial embolization, *USD* U.S. dollars

* Data are presented as means ± standard deviation

† The Charlson Comorbidity Index was scored based on each patient’s diagnoses [13]

‡ The number of TAEs performed at the hospital in the same fiscal year

### Incidence of early rebleeding and intestinal ischemic complications

The overall incidence of early rebleeding requiring intervention was 12%. Early rebleeding occurred in 11%, 14%, and 8% of patients in the coil, GS particle, and NBCA groups, respectively.

The overall incidence of intestinal ischemic complications was 1.0%. Ischemic intestinal complications occurred in 0.6%, 1.4%, and 2.1% of patients in the coil, GS particle, and NBCA groups, respectively.

### Logistic regression analyses

The results of bivariate logistic regression analyses are presented in Supplementary Tables S1 and S2. The results of multivariate logistic regression analyses are presented in Tables 2 and 3. With the coil as a reference, the adjusted ORs of early rebleeding were 1.38 (95% CI: 1.15–1.66, *p* = 0.001) for GS particles and 0.69 (95% CI: 0.50–0.95, *p* = 0.03) for NBCA. The adjusted ORs of intestinal ischemic complications were 2.64 (95% CI: 1.43–4.90, *p* = 0.002) for GS particles and 3.53 (95% CI: 1.72–7.22, *p* = 0.001) for NBCA. Thus, compared with coils, GS particles were associated with a significantly higher risk of both early rebleeding and intestinal ischemia, whereas NBCA was associated with a reduced risk of early rebleeding but an increased risk of intestinal ischemia.

**Table 2:** Results of multivariable logistic regression analysis for early rebleeding requiring intervention after TAE for CDB

Variable	Adjusted OR (95% CI)	*p*-value^a^
Embolic agent		
Coil	ref.	-
GS particle	1.38 (1.15–1.66)	0.001^b^
NBCA	0.69 (0.50–0.95)	0.03^b^
Age (years)	1.00 (0.99–1.01)	0.89
Male sex	1.13 (0.93–1.39)	0.22
Body mass index (kg/m^2^)		
18.5–24.9	ref.	-
< 18.5	1.06 (0.79–1.44)	0.69
≥ 25.0	1.10 (0.91–1.34)	0.32
Missing data	0.89 (0.64–1.25)	0.51
Smoking history		
Nonsmoker	ref.	-
Current and/or past smoker	0.94 (0.77–1.14)	0.53
Missing data	0.98 (0.76–1.26)	0.87
Charlson comorbidity index		
0	ref.	-
1	1.16 (0.89–1.53)	0.27
≥ 2	0.99 (0.80–1.22)	0.94
Admission due to CDB	0.93 (0.77–1.11)	0.42
Emergency admission	1.07 (0.77–1.47)	0.70
Ambulance transportation	1.04 (0.87–1.24)	0.69
Teaching hospital	1.03 (0.79–1.33)	0.83
Contrast-enhanced CT before TAE	0.98 (0.80–1.20)	0.85
Angiography before TAE	1.02 (0.63–1.63)	0.95
Colonoscopy before TAE	1.00 (0.84–1.19)	0.97
Colonoscopic hemostasis before TAE	1.35 (1.13–1.61)	0.001^b^
Transfusion before TAE	1.98 (1.58–2.48)	< 0.001^b^
Non-steroidal anti-inflammatory drugs usage before TAE	1.29 (0.99–1.67)	0.06
Antiplatelet or anticoagulant usage before TAE	0.99 (0.77–1.29)	0.96
Use of microcatheters	1.25 (0.79–1.98)	0.03^b^
Hospital volume*		
1	ref.	-
2	0.64 (0.50–0.81)	< 0.001^b^
3–4	0.64 (0.51–0.80)	< 0.001^b^
≥ 5	0.66 (0.52–0.84)	0.001^b^

*TAE* transcatheter arterial embolization, *CDB* colonic diverticular bleeding, *OR* odds ratio, *GS* gelatin sponge, *NBCA* n-butyl-2-cyanoacrylate

* Number of TAEs performed at the hospital in the same fiscal year

^a^ *p*-values were obtained using a generalized estimated equation

^b^ *p*-values indicate statistical significance (*p* < 0.05)

**Table 3:** Results of multivariable logistic regression analysis for intestinal ischemic complications after TAE for CDB

Variable	Adjusted OR (95% CI)	*p*-value^a^
Embolic agent		
Coil	ref.	-
GS particle	2.64 (1.43–4.90)	0.002^b^
NBCA	3.53 (1.72–7.22)	0.001^b^
Age (years)	1.01 (0.99–1.04)	0.30
Male sex	0.94 (0.50–1.76)	0.85
Body mass index (kg/m^2^)		
18.5–24.9	ref.	-
< 18.5	0.68 (0.24–1.93)	0.46
≥ 25.0	0.59 (0.29–1.20)	0.15
Missing data	0.53 (0.16–1.75)	0.30
Smoking history		
Nonsmoker	ref.	-
Current and/or past smoker	1.13 (0.60–2.14)	0.71
Missing data	0.91 (0.39–2.14)	0.83
Charlson comorbidity index		
0	ref.	-
1	1.67 (0.79–3.56)	0.18
≥ 2	0.60 (0.30–1.33)	0.21
Admission due to CDB	0.75 (0.43–1.36)	0.35
Emergency admission	0.46 (0.12–1.80)	0.27
Ambulance transportation	1.06 (0.60–1.86)	0.85
Teaching hospital	1.50 (0.53–4.25)	0.45
Contrast-enhanced CT before TAE	1.51 (0.69–3.31)	0.30
Angiography before TAE	1.31 (0.30–5.62)	0.72
Colonoscopy before TAE	0.62 (0.32–1.18)	0.15
Colonoscopic hemostasis before TAE	1.05 (0.56–1.96)	0.88
Transfusion before TAE	0.75 (0.41–1.39)	0.36
Non-steroidal anti-inflammatory drugs usage before TAE	2.58 (1.23–5.42)	0.01^b^
Antiplatelet or anticoagulant usage before TAE	0.55 (0.19–1.58)	0.27
Use of microcatheters	2.29 (0.31–16.98)	0.42
Hospital volume*		
1	ref.	-
2	1.05 (0.46–2.40)	0.91
3–4	0.97 (0.43–2.18)	0.93
≥ 5	1.50 (0.71–3.20)	0.29

*TAE* transcatheter arterial embolization, *CDB* colonic diverticular bleeding, *OR* odds ratio, *GS* gelatin sponge, *NBCA* n-butyl-2-cyanoacrylate

* Number of TAEs performed at the hospital in the same fiscal year

^a^ *p*-values were obtained using a generalized estimated equation

^b^ *p*-values indicate statistical significance (*p* < 0.05)

## Discussion

Using a nationwide inpatient database, we investigated 5625 patients who underwent TAE for CDB to evaluate differences in early rebleeding and intestinal ischemic complications between three embolic agents. The overall in-hospital incidences of early rebleeding requiring intervention and intestinal ischemic complications were 12% (*n* = 674) and 1.0% (*n* = 56), respectively. Compared to coils, early rebleeding occurred more often with GS particles (adjusted OR, 1.38; *p* = 0.001) and less often with NBCA (adjusted OR, 0.69; *p* = 0.02). The risk of intestinal ischemic complications was higher for both GS and NBCA (adjusted OR, 2.62; *p* = 0.002 and adjusted OR, 3.48; *p* = 0.001, respectively).

Studies on the incidence of early rebleeding and intestinal ischemic complications of TAE for CDB in the literature have been limited to those with up to 30 patients [6–8]. In contrast, expanding the scope of TAE for LGIB, 30% of which are estimated to be CDB [16–18], several studies involving approximately 50–150 patients have been reported [19–26]. Moreover, early rebleeding requiring therapeutic intervention reportedly occurred in 6.5–16.4% [20–25, 27]. Thus, our results were consistent with the previously reported studies. The incidence of intestinal ischemic complications ranges from 1.4 to 17.0% in previous studies [19–26]. Although they ranged widely according to the different definitions, the reported rates were generally higher than our results. Most minor intestinal ischemic complications are detected incidentally during follow-up endoscopy and resolve spontaneously [27, 28]. Therefore, our definition based on disease names may not have completely identified minor intestinal ischemic complications.

To the best of our knowledge, only one study has directly compared the outcomes of different embolic materials in TAE for LGIB [26]. In that study involving 141 patients, the use of glue showed a non-significant trend toward a higher incidence of ischemia-related bowel resection (*p* = 0.09). Theoretically, NBCA may be more hemostatic than coils due to its coagulation-independent embolic effect, whereas GS, a transient embolic material, may be less hemostatic; both materials may increase ischemic complications because of their high peripheral reachability [29–31]. This study presented real-world results reflecting these properties of embolic agents.

In this study, we evaluated various embolic materials (coils, NBCA, and GS particles) that were approved for TAE by the Japanese public health insurance system during the study period. However, in addition to the evaluated materials, other materials, such as polyvinyl alcohol particles and longer-polymerizing cyanoacrylates, are also used outside Japan for TAE [32]. Furthermore, we analyzed cases in which a single embolic material was used due to insufficient procedural details to interpret and compare the cases involving multiple embolic materials. Therefore, additional studies are required to investigate the efficacy and safety of other or multiple embolic materials.

Superselective embolization of a few vasa recta during TAE for LGIB has been suggested to reduce both rebleeding and ischemic complications. In a retrospective study involving 134 patients, embolization of fewer than three vasa recta was associated with reduced recurrent bleeding (adjusted OR, 0.295; *p* = 0.014) [33]. Moreover, in a study of TAE with NBCA, endoscopic and severe intestinal ischemia occurred in 6 and 0 of 14 patients, respectively, with one embolized vasa recta, whereas endoscopic intestinal ischemia and intestinal perforation occurred in 2 and 1 of 2 patients, respectively, with three or more embolized vasa recta [34]. Although we could not identify the number of embolized vasa recta in the current study, we incorporated microcatheter use to reflect the selectivity. Coils and NBCA are more commonly used in superselective embolization than GS particles [19, 21, 33]. A similar trend for embolic agents in patients with microcatheters was observed in this study. However, the multivariate analysis showed a higher risk of early rebleeding and intestinal ischemic complications in the GS particle group compared to the coil group. Nevertheless, further interventional studies considering angiographic features, such as the number of embolized vasa recta and the presence of collateral vessels, are required to validate our findings because microcatheter use may not totally represent selectivity.

The present study was a retrospective analysis of an administrative database. Thus, several inherent limitations should be recognized. First, the data may not be as accurate as that from studies that collect primary data. Additionally, a specific validation study for diagnosing CDB and intestinal ischemic complications has not been reported. Furthermore, detailed clinical information was not available, and outcomes were defined using procedures and diagnoses. Therefore, we were unable to identify patients with rebleeding or ischemia who did not receive additional procedures or had no diagnoses recorded, including those who died before thorough examination and treatment. These factors may lead to an underrepresentation of mild or fatal complications and introduce misclassification bias, thereby affecting the outcome estimates. Second, the locations and numbers of bleeding diverticula could not be determined. Although rebleeding from the same diverticulum has been reported in the short term [35], we could not confirm whether rebleeding originated from the treated diverticulum. This uncertainty may have led to the misclassification of new bleeding events from different diverticula as rebleeding from the treated site, potentially resulting in an overestimation of the true rebleeding rate. Third, we classified patients who underwent TAE without billing data for embolic agents into the GS particles group; however, some patients in the coil and NBCA groups may have received GS particles simultaneously, whereas some patients in the GS particle group could have been embolized with other agents. Such potential misclassification of embolic agents may have affected the estimation of intergroup differences. Fourth, a detailed evaluation of procedures could not be performed due to the lack of procedural parameters such as coil size, NBCA dilution ratio, and angiographic images. This procedural heterogeneity may confound the observed outcomes, as variations in embolization depth or technique may influence ischemic or rebleeding risks. Finally, excluding the aforementioned database-related limitations, there exists a limitation regarding generalizability to non-Japanese populations, as previous studies have shown that colonic diverticula location and bleeding incidence differ between races [1, 36, 37].

In conclusion, our investigation of 5625 patients who underwent TAE for CDB using a nationwide database found that, compared with coil embolization, GS particle embolization was associated with a higher risk of early rebleeding and intestinal ischemic complications, while NBCA embolization was associated with a lower risk of early rebleeding but a higher risk of intestinal ischemic complications. Thus, our results suggest that embolic materials should be selected based on individual patient profiles, such as ischemic risk and likelihood of rebleeding.

## Supplementary information

Supplementary information

## Abbreviations

CDB: Colonic diverticular bleeding
GS: Gelatin sponge
ICD-10: International Classification of Disease 10th Revision
LGIB: Lower gastrointestinal bleeding
NBCA: N-butyl-2-cyanoacrylate
OR: Odds ratio
TAE: Transcatheter arterial embolization
