# Supplementary material for: Association between embolic agent choice and complications after transcatheter arterial embolization for colonic diverticular bleeding
Source: Eur Radiol. 2025 Dec 19;36(5):3960–8. doi: 10.1007/s00330-025-12210-y (PMC13086735; doi:10.1007/s00330-025-12210-y)
Supplement: Supplementary file 1 — Supplementary information [file 330_2025_12210_MOESM1_ESM.pdf]

Association between embolic agent choice and complications after transcatheter arterial embolization for colonic diverticular bleeding

ELECTRONIC SUPPLEMENTARY MATERIAL

Eur Radiol (2025) Kubo T, Yamana H, Aso S, et al.

Table S1: Results of bivariate logistic regression analysis for early rebleeding requiring intervention after TAE for CDB

Variable	OR (95% CI)	*p*-value[a]
Embolic Agent		
Coil	ref.	-
GS Particle	1.30 (1.09–1.55)	0.004[b]
NBCA	0.69 (0.51–0.95)	0.02[b]
Age (y)	1.00 (0.99–1.01)	0.98
Male sex	1.11 (0.93–1.32)	0.27
Body mass index (kg/m^2)		
18.5–24.9	ref.	-
< 18.5	1.12 (0.84–1.50)	0.44
≥ 25.0	1.04 (0.86–1.25)	0.69
Missing data	0.89 (0.65–1.23)	0.49
Smoking history		
Nonsmoker	ref.	-
Current and/or past smoker	0.99 (0.83–1.18)	0.92
Missing data	1.00 (0.78–1.26)	0.97
Charlson Comorbidity Index		
0	ref.	-
1	1.24 (0.95–1.61)	0.11
≥ 2	1.01 (0.84–1.22)	0.93
Admission due to CDB	0.90 (0.76–1.06)	0.22
Emergency admission	0.92 (0.70–1.21)	0.55
Ambulance transportation	1.08 (0.92–1.27)	0.34
Teaching hospital	0.99 (0.77–1.28)	0.96
Contrast-enhanced CT before TAE	1.00 (0.82–1.21)	0.98

Angiography before TAE	1.16 (0.74–1.84)	0.52
Colonoscopy before TAE	1.17 (0.99–1.38)	0.07
Colonoscopic hemostasis before TAE	1.52 (1.28–1.79)	< 0.001[b]
Transfusion before TAE	2.01 (1.65–2.46)	< 0.001[b]
Non-steroidal anti-inflammatory drugs usage before TAE	1.35 (1.06–1.72)	0.02[b]
Antiplatelet or anticoagulant usage before TAE	1.20 (0.94–1.53)	0.15
Use of microcatheters	1.31 (0.85–2.04)	0.23
Hospital volume*		
1	ref.	-
2	0.64 (0.50–0.80)	< 0.001[b]
3–4	0.63 (0.50–0.78)	< 0.001[b]
≥ 5	0.58 (0.46–0.74)	< 0.001[b]

TAE transcatheter arterial embolization, CDB colonic diverticular bleeding, OR odds ratio, GS gelatin sponge, NBCA n-butyl-2cyanoacrylate

* Number of TAEs performed at the hospital in the same fiscal year.

[a] *p*-values were obtained using a generalized estimated equation.

[b] *p*-values indicate statistical significance ($p < 0.05$).

Table S2: Results of bivariate logistic regression analysis for intestinal ischemic complications after TAE for CDB

Variable	OR (95% CI)	*p*-value[a]
Embolic Agent		
Coil	ref.	-
GS Particle	2.27 (1.22–4.19)	0.009[b]
NBCA	3.31 (1.58–6.94)	0.001[b]
Age (y)	1.01 (0.99–1.04)	0.26
Male sex	0.82 (0.47–1.45)	0.50
Body mass index (kg/m^2)		
18.5–24.9	ref.	-
< 18.5	0.75 (0.26–2.14)	0.59
≥ 25.0	0.60 (0.30–1.21)	0.16
Missing data	0.63 (0.20–2.04)	0.45
Smoking history		
Nonsmoker	ref.	-
Current and/or past smoker	0.99 (0.55–1.78)	0.98
Missing data	0.86 (0.37–1.99)	0.72
Charlson Comorbidity Index		
0	ref.	-
1	1.68 (0.80–3.52)	0.17
≥ 2 ≤	0.62 (0.30–1.30)	0.21
Admission due to CDB	0.87 (0.50–1.52)	0.63
Emergency admission	0.51 (0.16–1.64)	0.26
Ambulance transportation	1.16 (0.68–1.97)	0.60
Teaching hospital	1.72 (0.61–4.88)	0.31
Contrast-enhanced CT before TAE	1.47 (0.70–3.09)	0.30

Angiography before TAE	1.39 (0.34–5.61)	0.65
Colonoscopy before TAE	0.61 (0.33–1.15)	0.13
Colonoscopic hemostasis before TAE	0.89 (0.49–1.61)	0.70
Transfusion before TAE	0.84 (0.48–1.47)	0.54
Non-steroidal anti-inflammatory drugs usage before TAE	1.96 (0.97–3.97)	0.06
Antiplatelet or anticoagulant usage before TAE	0.69 (0.25–1.89)	0.47
Use of microcatheters	2.25 (0.32–15.82)	0.42
Hospital volume*		
1	ref.	-
2	1.16 (0.51–2.64)	0.72
3–4	1.03 (0.46–2.33)	0.94
≥ 5	1.65 (0.78–3.52)	0.19

TAE transcatheter arterial embolization, CDB colonic diverticular bleeding, OR odds ratio, GS gelatin sponge, NBCA n-butyl-2cyanoacrylate

* Number of TAEs performed at the hospital in the same fiscal year.

[a] *p*-values were obtained using a generalized estimated equation.

[b] *p*-values indicate statistical significance ($p < 0.05$).
